# Trends in Pancreatic Cancer Incidence, Mortality and Net Survival: A Population-Based Analysis in Zurich (Switzerland), 1981–2021

**DOI:** 10.1007/s12029-026-01498-8

**Published:** 2026-05-28

**Authors:** Linda Vinci, Miriam Wanner, Andreas Wicki, Holger Moch, Dimitri Korol, Sabine Rohrmann

**Affiliations:** 1 Cancer Registry of the Cantons of Zurich, Zug, Schaffhausen, and Schwyz, University Hospital Zurich, Zurich, Switzerland; 2 Department of Medical Oncology and Hematology, University Hospital Zurich and University of Zurich, Zurich, Switzerland; 3 Department Pathology and Molecular Pathology, University Hospital Zurich and University of Zurich, Zurich, Switzerland; 4 Epidemiology, Biostatistics and Prevention Institute, University of Zurich, Zurich, Switzerland; 5 Division of Chronic Disease Epidemiology, Epidemiology, Biostatistics and Prevention Institute (EBPI), University of Zurich, Hirschengraben 84, Zurich, 8001 Switzerland

**Keywords:** Pancreatic cancer, Incidence, Mortality, Survival, Trend

## Abstract

**Background:**

Pancreatic cancer incidence is increasing worldwide while survival remains poor. The incidence varies significantly across different regions. The aim of the present study was to analyse incidence, mortality and survival of patients diagnosed with pancreatic cancer in the canton of Zurich, Switzerland.

**Methods:**

Population-based cancer registry data and cause of death statistics of the canton of Zurich from 1981 to 2021 were analysed. Age-standardised incidence and mortality rates per 100’000 person-years and net survival were calculated. Joinpoint regression analysis was conducted to identify significant changes in time trends while net survival was estimated using the Pohar-Perme method.

**Results:**

Age-standardised incidence rate of pancreatic adenocarcinomas (PAC) remained stable between 1981 and 2000 and then increased from 2000 to 2021, while pancreatic neuroendocrine tumours (PNET) incidence increased throughout and pancreatic cancers non histologically or cytologically confirmed (PC Non-HC) decreased. Overall age-standardised mortality rate remained stable. Five-year net survival was moderate for PNET (54.8%), but poor for PAC (4.8%) and PC Non-HC (1.2%). Among patients with PAC, survival declined with increasing age, earlier diagnosis year and advanced stage.

**Conclusions:**

Over the past 40 years, PNET incidence slowly increased. In contrast, PAC incidence significantly increased within the last 20 years. The underlying causes of the observed increase in PAC incidence might be attributable to special risk factors, e.g. obesity, but other risk factors should also be considered. Mortality rates of pancreatic cancer were stable and net survival was poor.

## Introduction

Pancreatic cancer is one of the most challenging malignancies in oncology. Despite accounting for only 3% of all cancer diagnoses worldwide, it is the sixth leading cause of cancer-related mortality globally [1]. Due to its aggressive behaviour, predominantly late-stage diagnosis, and limited therapeutic options, survival remained poor over the last decades [2]. Moreover, pancreatic cancer research has been historically underfunded, receiving less than 2% of cancer-related funding in Europe and ranking among the most underfunded cancers relative to its mortality burden in the USA [3].

Pancreatic cancers arise from exocrine or endocrine tissues of the pancreas. Pancreatic adenocarcinoma (PAC), which is the most common type, develops from exocrine cells and accounts for approximately 85% of cases. On the other hand, neoplasms derived from endocrine cells, known as pancreatic neuroendocrine tumours (PNET), represent fewer than 5% of pancreatic cancers [4]. While the precise aetiology of pancreatic cancer is unclear, several risk factors have been identified. Smoking, obesity, heavy alcohol consumption and chronic pancreatitis are the primary modifiable risk factors, while higher age and genetic predisposition are key non-modifiable risk factors [5]. Smoking and obesity was estimated to account for 10–30% of cases and genetic factors for less than 10% [6].

Global pancreatic cancer cases have more than doubled since 1990, with high-income countries experiencing the steepest increases. As life expectancy rises and healthcare access improves (which enables diagnosis and reporting) worldwide, absolute cases are expected to continue increasing, particularly among women and elderly populations. Current projections indicate a 95% increase in global pancreatic cancer cases from 2022 to 2050, with Asia, Europe, and North America expected to experience the largest absolute increases [7]. In contrast, global incidence and mortality rates increased until 2021 and are projected to decrease thereafter [8]. However, incidence and mortality rates vary significantly across different regions. The highest pancreatic cancer incidence rates worldwide are observed in Europe, North America and Australia/New Zealand [1]. In 2020, the global age-standardised incidence rate was 4.9 per 100’000 compared to 7.8 per 100’000 in Europe. Similarly, the global age-standardised mortality rate was 4.5. per 100’000, while the European rate reached 7.2 per 100’000. This difference between global and European rates probably results from variations in risk factor exposure but also better diagnostics in Europe [9].

Epidemiological data are essential for informing public health decision-making and resource allocation strategies. However, Switzerland, a high-income country with diverse cultural and linguistic regions, has limited comprehensive long-term analyses of pancreatic cancer incidence, mortality, and survival. Previous survival analyses from 1990 to 2010 have shown an increase in one-year relative survival rates at the national level [10]. To add knowledge we analysed incidence, mortality and net survival of patients diagnosed with PAC, PNET, and pancreatic cancers with no histological or cytological confirmation (PC Non-HC) in the canton of Zurich, Switzerland’s most populous canton between 1981 and 2021.

## Methods

### Cancer Registry Data

Population-based data of the cancer registry of the canton of Zurich was used to estimate incidence and survival. Patients with a primary pancreatic cancer diagnosed between 1981 and 2021 were included using the topography code C25 and all morphology codes for adenocarcinomas and neuroendocrine tumours of the International Classification of Diseases for Oncology. In addition, primary PC Non-HC between 1981 and 2021 were included. Cases based on death certificates only (DCO) were excluded.

The analyses could be stratified by cancer-stage only for the period between 2003 and 2021 due to a lack of systematic registration of the stages prior to 2003. Cancer-stage (IA, IB, IIA, IIB, III, IV and “unknown”) was defined according to the TNM classification of malignant tumours (TNM). Stages IA and IB as well as stages IIA and IIB were combined. TNM version 6 was applied to cases diagnosed between 2003 and 2009, version 7 to those diagnosed from 2010 to 2017 and version 8 to those diagnosed from 2018 to 2021. To define the TNM stage in the absence of pathological node variable (pN), the clinical node variable (cN) was used, and vice versa. In the absence of the pathological metastasis variable (pM), the clinical variable (cM) was applied, if available. When clinical metastasis stage was also unavailable, it was assumed to be zero. When neither pathological nor clinical data were available for node (pN and cN) and tumour (pT and cT), these were classified as missing. In the absence neoadjuvant therapy, pathological TNM stage was applied when available, otherwise clinical TNM stage was used for staging.

For our analysis, age was categorized into four groups: <60 years, 60–69 years, 70–79 years, and > 79 years. Year of diagnosis was categorized into four periods: 1981–1990, 1991–2000, 2001–2010, 2011–2021.

### Cause of Death Data

The canton of Zurich’s cause-of-death statistics provided by the Swiss Federal Office of Statistics was used to assess mortality between 1981 and 2021. It includes individuals residing in the canton on Zurich at the time of their death. The main cause of death was defined according to the eighth revision of the International Classification of Diseases (ICD-8) until 1994 and with the tenth revision (ICD-10) thereafter. Patients whose main cause of death was pancreatic cancer, defined by ICD-8 as 157 and by ICD-10 as C25, were included. In contrast to the incidence data, mortality data included overall pancreatic cancer cases and not only PAC, PNET and PC Non-HC as histological type and basis of diagnosis were not available in the mortality dataset.

### Statistical Analyses

Age-standardized incidence and mortality rates per 100,000 person-years were computed using midyear population estimates and the 1976 European Standard Population [11, 12]. Joinpoint regression analyses were conducted for incidence rates by histological type (PAC, PNET, PC non-HC), and for PAC by sex and age group. For mortality rates, analyses were performed overall by sex and age group. These analyses aimed to identify statistically significant changes in trend (joinpoints) and to calculate the annual percentage change (APC) for each regression segment and the average APC (AAPC) over the whole study period (1981–2021).

One and five-year net survival was estimated by histological type, and for PAC by sex, age at diagnosis, stage and year of diagnosis using the Pohar-Perme method [13]. Population life tables were provided by the National Agency for Cancer Registration.

Joinpoint regression analyses were conducted using the Joinpoint Regression Software Version 5.3.0 (November 2024 Statistical Research and Applications Branch, United States National Cancer Institute). All other analyses were performed using R Version 4.5.0 (2025 R Foundation for Statistical Computing).

### Ethics

Data collection in the cancer registry can be refused by patients at any time. To allow patients time to exercise this right, cases are registered three months after the initial report is received. If an objection is made after registration, all patient data except the information on the disease will be deleted immediately and no further data collection will occur [14]. The study was conducted in accordance with the Declaration of Helsinki (as revised in 2013). In accordance with the Swiss law on human research, approval from an ethics committee was not required because the data were anonymized.

## Results

### Cancer Registry Patient Characteristics

Between 1981 and 2021, 5256 patients were diagnosed with PAC, 361 with PNET and 1413 with PC non-HC in the canton of Zurich (Table 1). The majority of patients with PAC were in the 70–79 years age group (35.2%), those with PNET were predominantly < 60 years (41.0%) and those with PC non-HC were mostly > 79 years of age (58.9%). In all three subgroups, most cases were diagnosed between 2011 and 2021. Among patients with PAC, the majority were diagnosed with stage IV (47.2%). Patients with PNET were mostly diagnosed with either stages IA/IB (34.1%) or stage IV (33.7%). In patients with PC non-HC, the majority had an unknown stage (48.2%), followed by stage IV (41.1%).

**Table 1:** Number of pancreatic adenocarcinomas, pancreatic neuroendocrine tumours and pancreatic cancers non histologically or cytologically confirmed, 1981–2021, canton of Zurich, Switzerland

	PAC		PNET		PC Non-HC	
	*N*	%	*N*	%	*N*	%
Sex						
Men	2665	50.7	206	57.1	547	38.7
Women	2591	49.3	155	42.9	866	61.3
Age at diagnosis in years						
< 60	930	17.7	148	41.0	53	3.8
60–69	1416	26.9	87	24.1	140	9.9
70–79	1851	35.2	90	24.9	388	27.5
> 79	1059	20.1	36	10.0	832	58.9
Year of diagnosis						
1981–1990	965	18.4	39	10.8	283	20.0
1991–2000	905	17.2	65	18.0	299	21.2
2001–2010	1220	23.2	74	20.5	364	25.8
2011–2021	2166	41.2	183	50.7	467	33.1
Basis of diagnosis						
Clinical	0	0.0	0	0.0	362	25.6
Clinical investigation	0	0.0	0	0.0	965	68.3
Specific tumour markers	0	0.0	0	0.0	86	6.1
Cytology	1687	32.1	42	11.6	0	0.0
Histology of a metastasis	620	11.8	32	8.9	0	0.0
Histology of a primary tumour	2949	56.1	287	79.5	0	0.0
Topography						
Head of pancreas (C250)	2593	49.3	102	28.3	727	51.5
Body of pancreas (C251)	547	10.4	37	10.2	77	5.4
Tail of pancreas (C252)	633	12	95	26.3	82	5.8
Pancreatic duct (C253)	10	0.2	0	0.0	0	0.0
Endocrine pancreas (C254)	2	0.0	17	4.7	0	0.0
Other parts of pancreas (C257)	10	0.2	0	0.0	0	0.0
Overlapping lesion of pancreas (C258)	119	2.3	10	2.8	20	1.4
Pancreas, unspecified (C259)	1342	25.5	100	27.7	507	35.9
Stage^a^						
IA and IB	139	4.3	84	34.1	23	3.0
IIA and IIB	663	20.7	36	14.6	37	4.9
III	217	6.8	4	1.6	21	2.8
IV	1509	47.2	83	33.7	313	41.1
Unknown	672	21.0	39	15.9	367	48.2
Grade						
1 (well differentiated)	201	3.8	115	31.9	0	0
2 (moderately differentiated)	1139	21.7	83	23	0	0
3 (poorly differentiated)	1462	27.8	56	15.5	0	0
4 (undifferentiated)	1	0	1	0.3	0	0
Unknown	2453	46.7	106	29.4	1413	100

Abbreviations: *PAC*, Pancreatic adenocarcinoma; *PNET*, Pancreatic neuroendocrine tumours; *PC Non-HC*, Pancreatic cancers non histologically or cytologically confirmed

^a^Cancer-stage analysis was performed for the period between 2003 and 2021 because the stages were not registered systematically prior to 2003

### Incidence Trends

**Fig. 1:**
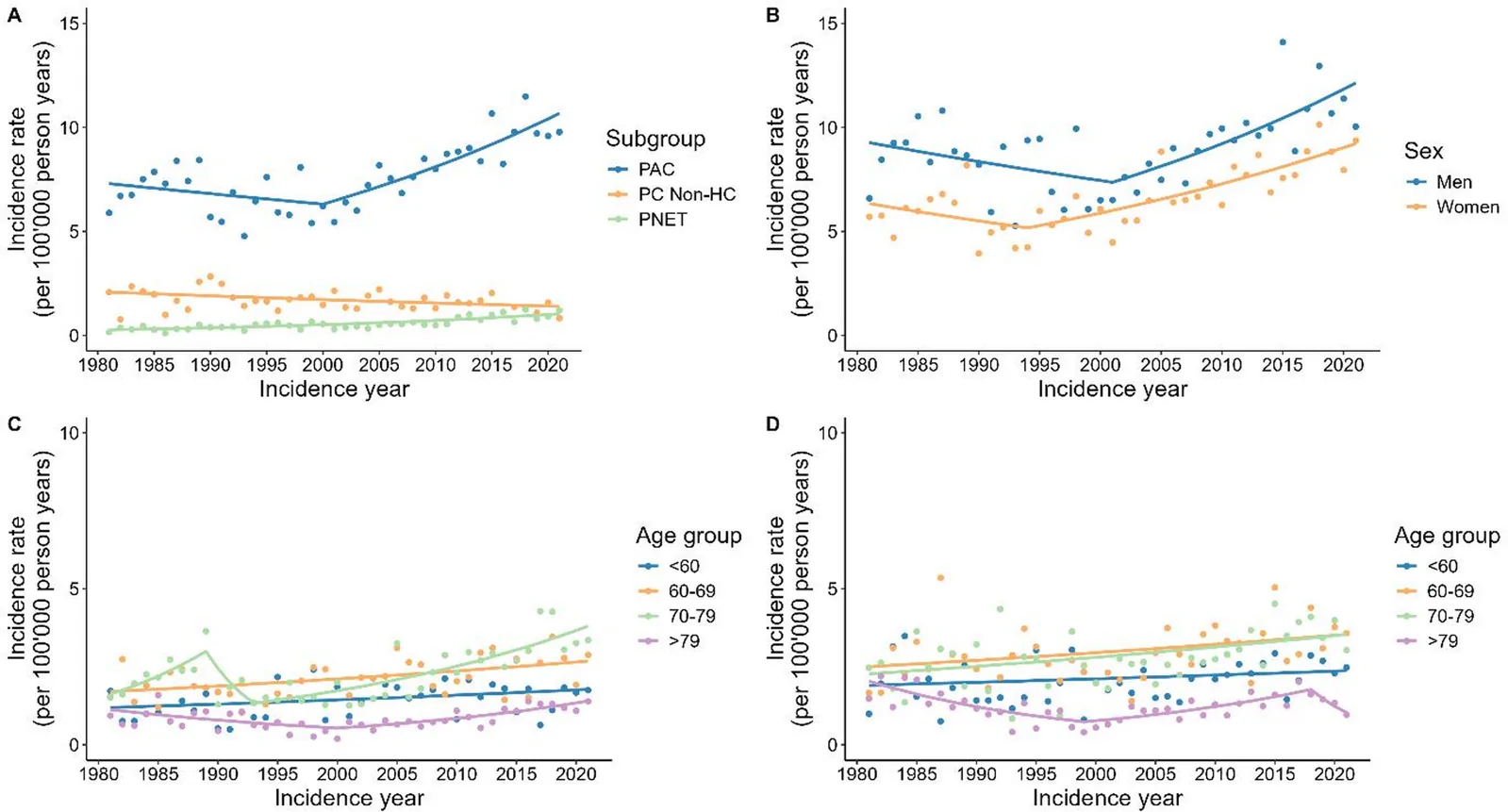
Age-standardised incidence rates of pancreatic cancer by histological type (**A**) and pancreatic adenocarcinomas (PAC) by sex (**B**), and by age group in women (**C**) and men (**D**), canton of Zurich, Switzerland, 1981–2021. Joinpoint regression lines were added. Abbreviations: PAC, Pancreatic adenocarcinoma; PNET, Pancreatic neuroendocrine tumours; PC Non-HC, Pancreatic cancers non histologically or cytologically confirmed

Age-standardised incidence rate of PAC remained stable between 1981 and 2000 and then increased from 2000 to 2021 (Table 2; Fig. 1A). In contrast, the incidence rate of PNET increased throughout the entire period while that of PC non-HC decreased. However, PAC occurred at a significantly higher rate than PNET and PC Non-HC.

**Table 2:** Trends of pancreatic cancer incidence and mortality rates, 1981–2021, canton of Zurich, Switzerland

	Entire period (1981–2021)	Period 1		Period 2		Period 3	
	AAPC (95% CI)	Period	APC (95% CI)	Period	APC (95% CI)	Period	APC (95% CI)
Incidence							
PAC	1.0 (0.4 to 1.5) *	1981–2000	-0.8 (-4.1 to 0.5)	2000–2021	2.5 (1.7 to 4.7) *		
PC Non-HC	-1.0 (-1.7 to -0.2) *						
PNET	3.4 (2.6 to 4.7) *						
PAC men by age							
< 60	0.6 (-0.2 to 1.5)						
60–69	0.9 (0.2 to 1.7) *						
70–79	1.1 (0.5 to 2.1) *						
> 79	-1.7 (-3.0 to 0.0)	1981–1999	-5.5 (-11.5 to -0.7) *	1999–2018	4.7 (-7.4 to 30.7)	2018–2021	-16.5 (-37.3 to 2.9)
Total	0.7 (0.0 to 1.4)	1981–2001	-1.1 (-5.9 to 0.3)	2001–2021	2.5 (1.4 to 6.0) *		
PAC women by age							
< 60	1.0 (0.1 to 2.3) *						
60–69	1.1 (0.5 to 2.0) *						
70–79	2.2 (1.3 to 3.5) *	1981–1989	8.0 (-0.1 to 30.9)	1989–1993	-18.3 (-31.0 to 2.3)	1993–2021	3.8 (2.8 to 5.4) *
> 79	0.6 (-0.5 to 1.8)	1981–2000	-3.8 (-9.8 to -0.7) *	2000–2021	4.7 (2.9 to 9.3) *		
Total	0.9 (0.3 to 1.7) *	1981–1994	-1.6 (-10.0 to 0.9)	1994–2021	2.2 (1.5 to 4.8) *		
Mortality							
Total	0.3 (-0.2 to 0.8)	1981–1989	2.1 (-1.4 to 9.9)	1989–1992	-9.7 (-13.1 to 3.8)	1992–2021	0.8 (-0.1 to 1.5)
Men by age							
< 60	-0.6 (-1.4 to 0.4)						
60–69	-0.5 (-1.2 to 0.3)						
70–79	0.0 (-0.6 to 0.6)						
> 79	-0.2 (-0.9 to 0.8)						
Total	-0.3 (-0.8 to 0.3)	1981–2002	-1.3 (-6.2 to -0.4) *	2002–2021	0.8 (-0.1 to 6.0)		
Women by age							
< 60	0.0 (-1.0 to 1.2)						
60–69	0.2 (-0.5 to 1.1)						
70–79	0.1 (-0.7 to 1.0)	1981–1999	-3.0 (-8.8 to -0.8) *	1999–2021	2.7 (1.2 to 6.5) *		
> 79	0.3 (-0.6 to 1.3)	1981–1995	-2.8 (-13.7 to 0.3)	1995–2021	2.0 (1.1 to 5.8) *		
Total	0.1 (-0.4 to 0.9)	1981–1994	-1.6 (-10.6 to 0.4)	1994–2021	1.0 (0.3 to 5.4) *		

Abbreviations: *AAPC*, Average Annual Percent Change; *APC*, Annual Percent Change; *PAC*, Pancreatic adenocarcinoma; *PC Non-HC*, Pancreatic cancers non histologically or cytologically confirmed; *PNET*, Pancreatic neuroendocrine tumours

* Indicates that the APC is significantly different from zero at the alpha = 0.05 level

Subgroup analysis of PAC revealed that the incidence rate was higher in men than in women (Table 2; Fig. 1B. While the incidence rate began to increase in women from 1994 onward, in men it only started to increase from 2001 onward. Over the entire study period, the increase in incidence rate was statistically significant in women but not in men Table 2). In women, an increase in incidence rate over the whole study period was observed across all age groups, except for the oldest group (> 79 years), which remained stable (Table 2; Fig. 1C). The 70 to 79 years age group showed the largest increase over the whole study period and had two joinpoints in 1989 and 1993. The two youngest age groups had no joinpoints, while the oldest age group had a joinpoint in 2000 where the trend shifted from decreasing to increasing. In men, however, the PAC incidence rates over the whole study period remained stable for the youngest (< 60 years) and oldest age groups (> 79 years), while the rates in the two intermediate age groups (60–69 and 70–79 years) increased (Table 2; Fig. 1D). Two joinpoints were identified only in the > 79 years age group.

### Mortality Trends

**Fig. 2:**
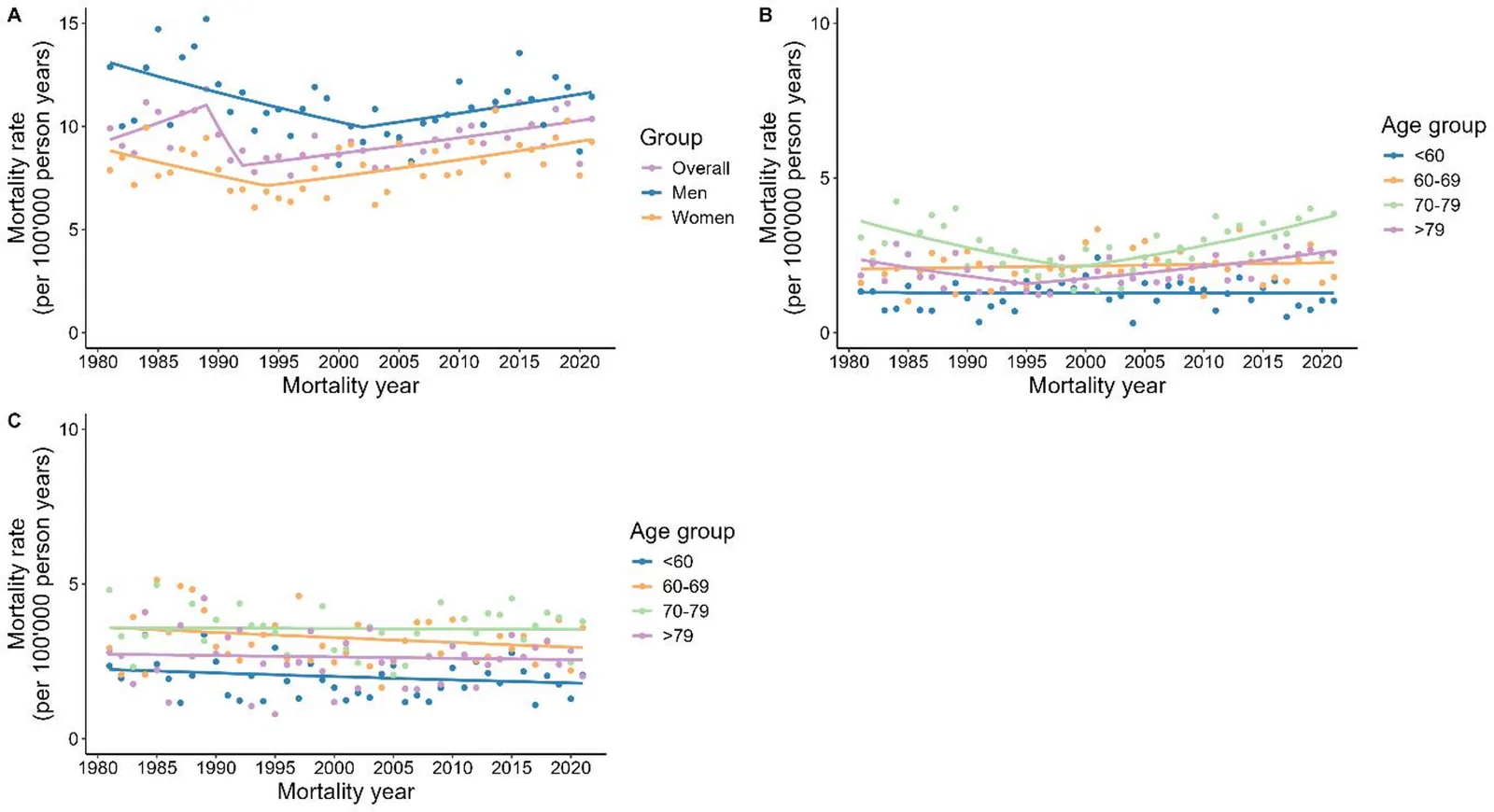
Age-standardised mortality rates of pancreatic cancer by sex (**A**) and by age group in women (**B**) and men (**C**), canton of Zurich, Switzerland, 1981–2021 Joinpoint regression lines were added

The overall age-standardised mortality rate remained stable throughout the study period, also stratified by sex (Table 2; Fig. 2A). Although joinpoints were identified in 1989 and 1992, no statistically significant changes were observed at these points nor across the entire study period. However, in women the mortality rate remained stable between 1981 and 1994 and then increased until 2021 (Table 2; Fig. 2A). On the other hand, in men the mortality rate decreased between 1981 and 2002 and then remained stable until 2021. However, in men the mortality rate was higher than in women (Fig. 2A).

In women, the trend in the 70–79 years age group shifted from a decreasing to an increasing trend in 1999, while the trend in the > 79 years age group changed from a stable to an increasing trend in 1995 (Table 2; Fig. 2B). The trend in the two younger age groups remained stable. However, over the entire study period the trend in all age groups remained stable. In contrast, in men, mortality remained stable across all age groups, with no joinpoints identified (Table 2; Fig. 2C).

### Survival

**Fig. 3:**
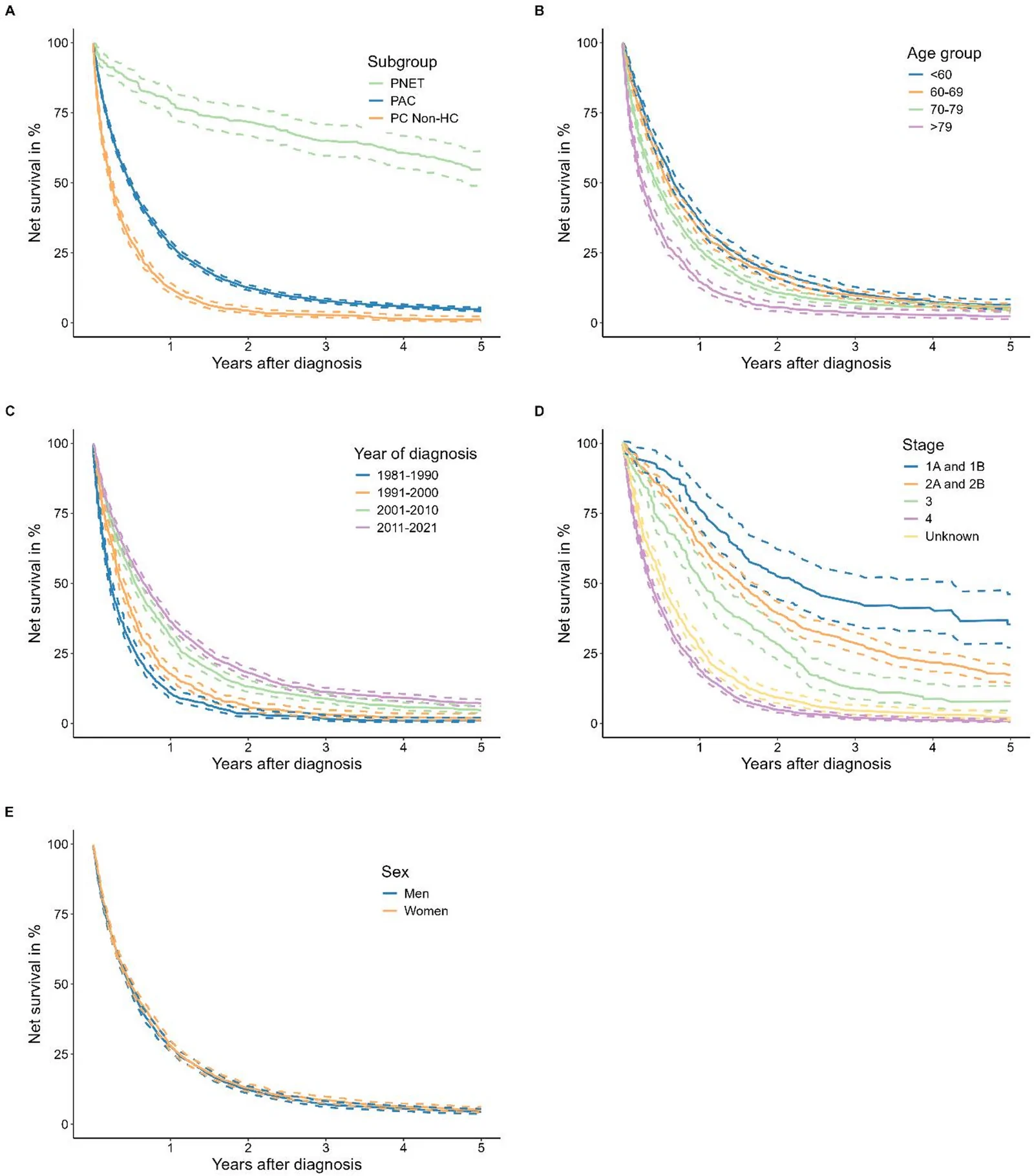
Net survival of pancreatic cancer by histological subgroup (**A**) and pancreatic adenocarcinoma by age group (**B**), year of diagnosis (**C**), stage (**D**) and sex (**E**), canton of Zurich, Switzerland, 1981–2021. Cancer-stage analysis was performed for the period between 2003 and 2021 because the stages were not registered systematically prior to 2003. Dashed lines correspond to the 95% confidence intervals. Abbreviations: PAC, Pancreatic adenocarcinoma; PNET, Pancreatic neuroendocrine tumours; PC Non-HC, Pancreatic cancers non histologically or cytologically confirmed

Net survival varied considerably across groups. One-year and five-year survival were moderate for PNET (78.9% [95% CI: 74.5% to 83.6%] and 54.8% [95% CI: 49.0% to 61.3%], respectively), but poor for PAC (27.9% [95% CI: 26.6% to 29.2%] and 4.8% [95% CI: 4.1% to 5.5%], respectively) and PC non-HC (12.3% [95% CI: 10.7 to 14.3%] and 1.2% [95% CI: 0.6 to 2.3%], respectively) (Fig. 3A). Due to insufficient case numbers for PNET and PC non-HC, subgroup analyses were performed only for PAC, revealing variations in survival by age, year of diagnosis, and stage.

At one and five years after diagnosis, survival was 36.5% (95% CI 33.5% to 39.8%) and 6.5% (95% CI: 5.0% to 8.4%) for patients with PAC aged < 60 years compared to 14.8% (95% CI: 12.6% to 17.4%) and 2.4% (95% CI: 1.4% to 4.2%) for those > 79 years (Fig. 3B. Similarly, patients with PAC diagnosed between 2011 and 2021 had a one- and five- year survival of 36.5% (95% CI: 34.5% to 38.6%) and 7.3% (95% CI: 6.1% to 8.7%) compared to 10.9% (95% CI: 9.0% to 13.2%) and 1.1% (95% CI: 0.5% to 2.1%) for those diagnosed between 1981 and 1990 (Fig. 3C). The most pronounced difference was observed by stage: patients with stage I had one- and five-year survival of 76.7% (95% CI: 69.6% to 84.6%) and 35.4% (95% CI: 27.1% to 46.2%) compared to 19.2% (95% CI: 17.3%. to 21.3%) and 0.9% (95% CI: 0.05% to 1.6%) for stage IV (Fig. 3D. No difference in net survival was observed between sexes (Fig. 3E). Men had one- and five-year survival of 27.7% (95% CI: 26.0% to 29.6%) and 4.5% (95% CI: 3.7% to 5.5%) compared to 28.0% (95% CI: 26.2% and 29.9%) and 5.0% (95% CI: 4.2% to 6.1%) for women.

## Discussion

Age-standardised incidence rate of PAC remained stable between 1981 and 2000 and then increased from 2000 to 2021, while the incidence rate of PNET increased throughout the entire period and that of PC non-HC decreased. In contrast, the overall age-standardised mortality rate remained stable throughout the study period 1981–2021. One-year and five-year net survival was moderate for PNET but poor for both PAC and PC non-HC. Among patients with PAC, survival declined with increasing age, earlier diagnosis year and advanced stage.

### Incidence Trends by Histological Type

The observed trends by histological type are in line with other European findings. In Girona, Spain, from 1993 to 2015, non-pancreatic neuroendocrine tumours (non-PNET) incidence rate increased, PNET incidence rate remained stable and the incidence rate of PC Non-HC decreased over time [15]. The decrease in PC non-HC incidence rate probably reflects the adoption and increasing utilisation of advanced diagnostics methods and more frequent use of biopsies [15]. The increase in non-PNET incidence rates over time is mainly due to the aging population, but also the increased exposure to risk factors (high BMI, diabetes) and improvements in diagnostic capabilities and reporting contribute to this upward trend [16–20]. In contrast, the observed rise in PNET incidence rates may be partly attributable to an increase in abdominal computed tomography (CT) utilisation. However, data on CT scan frequency over time are scarce for Switzerland. Aroua et al. [21] estimated a frequency of 19.7 per 1,000 inhabitants for full abdominal CT in 2008, while Viry et al. [22] reported an abdominal CT frequency of 23.9 per 1,000 inhabitants in 2018. Although these numbers suggest a slight increase over time, direct comparison must be interpreted with caution, as the estimates derive from two independent studies that differ in methodology.

### PAC Incidence Trends

In line with our findings, previous studies have documented higher incidence rates of PAC among men compared with women [23–25]. The mechanisms underlying these sex- differences are unknown but they may be attributable to lifestyle-related and environmental risk factors, including occupational exposures such as nickel [9]. We observed a statistically significant increase in PAC incidence over time among women, whereas no significant increase was detected among men. In contrast, other population-based studies have reported increasing incidence rates of PAC or pancreatic ductal adenocarcinoma (which constitutes the vast majority of PAC) in both sexes over time [20, 23, 24, 26]. However, these studies examined different and generally shorter time periods than the present study, which may explain the different trends observed. Furthermore, the increase in men that we observed over the entire study period is non-significant (AAPC 0.7, 95% CI: 0.0 to 1.4) but a statistically significant increase was observed in men from 2001 to 2021.

A Dutch study observed increasing incidence rates across all age groups from 1997 to 2016 [23]. In contrast, our study identified a rise in PAC incidence rates among women < 60 years old, but not in men of the same age. Similar sex-specific trends in younger populations have been documented in the United States. Jiang et al. found that incidence rates of pancreatic ductal adenocarcinoma increased among women < 55 years old between 2000 and 2018, whereas rates in men < 55 years old remained stable [26]. The most pronounced relative increase was observed in the youngest age group (15–34 years) [27]. The underlying causes of the observed increase in PAC incidence among younger women are unclear, though they are probably associated with different exposure to risk factors [26], particularly tobacco consumption, which is a primary risk factor for early-onset pancreatic cancer [28]. In Switzerland, tobacco smoking prevalence in women remained stable between 1992 and 2022 while in men it decreased from 37% to 27% over the same period [29]. Given the 10- to 50-year latency period for pancreatic cancer development [30], the smoking trends may partially explain the sex-specific PAC incidence trends observed from 2000 onwards.

### Mortality Trends

Although PAC and PNETincidence rates increased and survival remains poor, overall mortality rates did not mirror the incidence trends but remained stable. In contrast, other countries observed an increase in mortality rates for men and women [31], particularly among women since 1991. Our findings align with this pattern for women, showing an increase in mortality from 1994 to 2021. Consistent with European countries [9, 32, 33], mortality rates were higher in men than in women, reflecting the higher incidence rates observed in men.

PAC incidence rates in women increased from 1993 onwards in the 70–79 age group and from 2000 onwards in those aged > 79 years. Corresponding increases in pancreatic cancer mortality were observed, with rates in the 70–79 age group rising from 1999 onwards and rates in those aged > 79 years rising from 1995 onwards. In contrast, among women aged < 60 and 60–69 years, PAC incidence rates increased throughout the study period while pancreatic cancer mortality rates remained stable. In men, pancreatic cancer mortality rates remained stable across all age groups, despite of variations in age-specific incidence rates. These discordances between sex and age-specific incidence and mortality trends have been observed in other countries [31]. The continued increase in pancreatic cancer mortality among older women (≥ 70 years), despite therapeutic advances, requires further investigation.

### Survival Analyses by Tumour Type

In accordance with other studies, PNET had the best survival while PAC had very poor survival [15, 34]. We observed slightly lower survival rates than other studies, though regional variation has been documented. While we observed a 5-year survival of 54.8% (95% CI: 49.0% to 61.3%) for PNET, Garcia et al. [15] reported 61.45% for PNET (95% CI: 47.47 to 79.55). Stang et al. [34] found small regional differences within Germany: PNET survival was 62.3% in North Rhine-Westphalia and 53.7% in Saarland. For PAC Stang et al. [34] reported 5-year survival of 11.3% (standard error [SE] 0.4) in North Rhine-Westphalia and 10.2% (SE 1.5) in Saarland, compared to our observed net survival of 4.8% (95% CI: 4.1% to 5.5%). However, comparability with Stang et al. [34] is limited as they used the method by Ederer to calculate the relative survival.

### PAC Survival Analyses

In contrast to Garcia et al. [15], we observe no difference in one- and five-year survival between sexes for PAC. Garcia et al. [15] found slightly better 5-year survival in men than in women (4.75% versus 2.9%) for non-PNETs and non-HC PC (of which the vast majority are likely PAC). However, our findings align with Yasinzai et al. [35] who also reported similar survival between sexes at both one-year (women: 30.7% versus men: 29.8% ) and 5-years (women: 5.4% versus 5.1%).

Consistent with Garcia et al. [15] for non-PNETs and non-HC PC, we observed declining PAC survival with increasing age. However, in the present study the difference was most pronounced at one year, and the difference diminished at five years.

Survival declined with advancing stage, consistent with other studies [15, 34]. While Garcia et al. [15] observed the best survival for stage IIA without considering stage IA and IB, we found the best survival for stage IA and IB. Bengtsson et al. [36] similarly reported declining actual survival with advanced stage.

In line with other studies [15, 36] we observed improved survival in more recent diagnosis years.

### Strengths and Limitations

This study has several strengths. First, we used population-based data from the cancer registry of the canton of Zurich with good quality and completeness [37]. Second, the long observation period of 40 years enabled comprehensive temporal trend analyses. Third, we included all three major pancreatic cancer subtypes. To our knowledge, this is the first study to provide net survival estimates for PAC, PNET, and PC non-HC using Swiss cancer registry data.

However, several limitations must be acknowledged. Due to insufficient case numbers, subgroup analyses by sex, age, stage, and year of diagnosis could only be conducted for PAC; sample sizes for PNET and PC non-HC were too small. Mortality data did not allow stratification by subtype (PAC, PNET, and PC non-HC). Important risk factors for pancreatic cancer, including tobacco consumption, obesity, heavy alcohol consumption, and genetic predisposition, were not available in the cancer registry data. Additionally, stage information was only systematically recorded from 2003 onwards, limiting stage-stratified analyses to the more recent period.

## Conclusions

Age-standardised incidence rate of PAC remained stable between 1981 and 2000 and then increased from 2000 to 2021, while the incidence rate of PNET increased throughout the entire period and that of PC non-HC decreased. In contrast, the overall age-standardised mortality rates remained stable throughout the entire study period. Survival for PAC was poor, particularly among older patients, those diagnosed in earlier calendar years and those with advanced stage. These findings highlight the need for improved early detection methods and more effective therapeutic approaches.

## Data Availability

The datasets generated during and/or analysed during the current study are available from the corresponding author on reasonable request. The datasets are not publicly available due to data protection reasons.
